# Clustering of energy balance-related behaviors and parental education in European children: the ENERGY-project

**DOI:** 10.1186/1479-5868-10-5

**Published:** 2013-01-15

**Authors:** Juan M Fernández-Alvira, Ilse De Bourdeaudhuij, Amika S Singh, Frøydis N Vik, Yannis Manios, Eva Kovacs, Natasa Jan, Johannes Brug, Luis A Moreno

**Affiliations:** 1 GENUD (Growth, Exercise, Nutrition and Development) Research Group. Faculty of Health Sciences, University of Zaragoza, Zaragoza 50009, Spain; 2 Department of Movement and Sport Sciences, Ghent University, Ghent, Belgium; 3 Department of Public and Occupational Health and EMGO Institute for Health and Care Research, VU University Medical Center, Amsterdam, the Netherlands; 4 Department of Public Health, Sport and Nutrition, University of Agder, Kristiansand, Norway; 5 Department of Nutrition and Dietetics, Harokopio University, Athens, Greece; 6 Department of Pediatrics, Pecs University, Pecs, Hungary; 7 Slovenian Heart Foundation, Ljubljana, Slovenia; 8 Department of Epidemiology and Biostatistics and EMGO Institute for Health and Care Research, VU University Medical Center, Amsterdam, the Netherlands

**Keywords:** Energy balance-related behaviors, ENERGY, Children, Parental education, Cluster analysis

## Abstract

**Background:**

Recent research and literature reviews show that, among schoolchildren, some specific energy balance-related behaviors (EBRBs) are relevant for overweight and obesity prevention. It is also well known that the prevalence of overweight and obesity is considerably higher among schoolchildren from lower socio-economic backgrounds. This study examines whether sugared drinks intake, physical activity, screen time and usual sleep duration cluster in reliable and meaningful ways among European children, and whether the identified clusters could be characterized by parental education.

**Methods:**

The cross-sectional study comprised a total of 5284 children (46% male), from seven European countries participating in the ENERGY-project (“EuropeaN Energy balance Research to prevent excessive weight Gain among Youth”). Information on sugared drinks intake, physical activity, screen time and usual sleep duration was obtained using validated self-report questionnaires. Based on these behaviors, gender-specific cluster analysis was performed. Associations with parental education were identified using chi-square tests and odds ratios.

**Results:**

Five meaningful and stable clusters were found for both genders. The cluster with high physical activity level showed the highest proportion of participants with highly educated parents, while clusters with high sugared drinks consumption, high screen time and low sleep duration were more prevalent in the group with lower educated parents. Odds ratio showed that children with lower educated parents were less likely to be allocated in the active cluster and more likely to be allocated in the low activity/sedentary pattern cluster.

**Conclusions:**

Children with lower educated parents seemed to be more likely to present unhealthier EBRBs clustering, mainly characterized by their self-reported time spent on physical activity and screen viewing. Therefore, special focus should be given to lower educated parents and their children in order to develop effective primary prevention strategies.

## Background

Despite a leveling-off of obesity prevalence in some countries in the last years, childhood obesity still shows an unacceptable high prevalence [1], with secular trends to higher fat mass and more central fat distribution, even in non-obese children and adolescents [2]. Moreover, evidence shows that overweight and obesity track from childhood to adulthood [3,4].

Even if genetic factors may influence the susceptibility of some individuals to gain weight [5], there is a general consensus that lifestyle factors are driving the obesity epidemic [6]. Recent research and literature reviews show that, among schoolchildren, some specific energy balance-related behaviors (EBRBs) are at least associated with overweight and obesity prevalence and may be important for obesity prevention [7-11]. These behaviors comprise, among others, the intake of sugared drinks, screen viewing behavior (TV viewing and sedentary computer activities) and regular physical activities, like active commuting to school, participation in sports and recreational physical activity. In addition, recent evidence suggests that sleeping habits may also be relevant for energy balance [12,13].

It is also well known that the prevalence of overweight and obesity is considerably higher among youth from lower socio-economic backgrounds [14-18].

Parental education has been associated with several EBRBs in children and adolescents, showing higher sugared drinks consumption [19,20], lower physical activity levels [21] and higher sedentary behavior levels [22] among children with lower educated parents, while mixed results have been found regarding the association between parental education and sleep duration [23-25]. The majority of the literature focused on each of these EBRBs separately, sometimes also including reports on its socio-demographic correlates [22,26-28]. However, there is little information about the co-occurrence of these EBRBs and the association with socio-demographic factors. Specifically cross-European studies providing insight about EBRBs clustering and their socio-demographic correlates are lacking.

EBRBs clustering refers to a combination of behaviours which is more prevalent than expected based on the prevalence of the separate behaviours [29]. The potential synergy between EBRBs should be taken into account in obesity prevention interventions, as the combination of several unhealthy behaviors could lead to a multiplication of the risk. Furthermore, a better insight in the clustering of multiple behaviors in relation to socio-demographic correlates could help to identify subgroups at increased risk in developing overweight and obesity. Since childhood and adolescence are critical periods during lifetime in adopting health behaviors, the study of multiple health indices should be a public health priority.

In addition to the recently published report on EBRBs differences by Brug et al. [26], in which children of lower educated parents reported less favorable intakes regarding softdrinks and fruitjuice, higher total screentime and lower participation in sports than those from higher educated parents, cluster analysis was applied in this study.

It allows to specifically detect co-ocurrence of risky EBRBs levels. Additionally, it is possible to evaluate if these combinations of risky EBRBs are more prevalent in some groups characterized by parental education level.

More precisely, the first aim of the analysis was to examine whether EBRBs assessed in the cross-European study (i.e. sugared drinks intake, physical activity, screen time and usual sleep duration) cluster in a reliable and meaningful way among European children, characterized by healthy or unhealthy EBRBs combinations. The second aim was to investigate whether these identified clusters could be characterized by parental education, taking into account other important correlates, like gender and Body Mass Index (BMI).

## Methods

### Study population

Data were obtained from the cross sectional study of the “EuropeaN Energy balance Research to prevent excessive weight Gain among Youth” (ENERGY) project. This cross-sectional study was carried out between March and July 2010 in Belgium, Greece, Hungary, the Netherlands, Norway, Slovenia and Spain, among pupils in the final years of primary education (aged 10–12 years). The aim of the survey was to provide up to date information on the prevalence of overweight and obesity, on the most important EBRBs and their social, cognitive and school environmental determinants.

Based on previous cross-European studies, a minimum sample of 1000 schoolchildren per country and one parent/caretaker for each child were aimed for [30]. The schools were randomly selected concerning the degree of urbanization of the different provinces and the socioeconomic status (SES) of the different areas within the selected provinces. Samples were national representative in Greece, Hungary, the Netherlands and Slovenia. In Spain, schools of the region of Aragón were selected; Belgium selected schools from Flanders and Norway selected schools from the southern regions of the country [31].

A description of the rationale, design, procedures and methodology of the ENERGY school-based survey is published elsewhere [32]. A first paper on prevalence of overweight, obesity and engagement in different EBRBs as well as differences in these prevalence according to parental education, has been published elsewhere [26]. The studies were approved by the corresponding local ethics committees.

Belgium: The Medical Ethics Committee of the Ghent University Hospital

Greece: The Bioethics Committee of Harokopio University

Hungary: The Scientific and Ethics Committee of Health Sciences Council.

The Netherlands: The Medical Ethics Committee of the VU University medical center

Norway: The National Committees for Research Ethics in Norway

Slovenia: The National Medical Ethics Committee of the Republic of Slovenia

Spain: The Clinical Research Ethics Committee of the Government of Aragon

School recruitment letters were sent to the headmasters or principals of the sampled schools, and after school’s agreement, parents were asked for written consent for their child’s and own voluntary participation.

### Data collection

Information on children’s EBRBs and parental education was obtained using self-reported questionnaires. Anthropometric measures were performed by trained researchers/research assistants following a according to standardized protocols. The children completed questionnaires and anthropometric measurements were performed during school time.

#### Energy balance-related behaviors

Children provided data on dietary, physical activity and screen viewing behaviors via the child questionnaire, while sleep duration was reported by the parents. Both children and parental questionnaires showed a good test-retest reliability and moderate to good construct validity for the large majority of items [33].

**Dietary behaviors **Intakes of soft drinks and fruit juices were assessed with two food frequency questions. Children were asked how many days per week they drank the beverage, answering on a seven-point scale from never to more than once every day. Afterwards they were asked to indicate how much they drank by ticking the number of glasses or small bottles (e.g. 250 ml), cans (i.e. 330 ml) and large bottles (i.e.500 ml) for soft drinks, or glasses/small cartons (i.e.250 ml) and regular cartons (330 ml) for fruit juices. The questionnaire included pictures of the serving sizes. Mean intake in milliliter (ml) per day was calculated from these two questions. In addition, children were asked to fill in how much of the beverages they had consumed on the day before, following the same classification. For the purposes of this analysis, ml/day of sugared drinks (soft drinks + fruit juices) were taken into account.

**Physical activity behaviors **Active transportation to school was assessed by two questions about how many days per week the child cycled and/or walked to school, ranging from never to 5 days/week, and two questions on the duration of biking or walking to school, with 4 answer categories ranging from 1–5 minutes to more than 15 minutes. Total active transportation time per week was calculated by adding up total bike and walk times and multiplying the number of days with the mean time of the answering category times 2. Organized sports participation was assessed with specific questions about how many hours per week children participated in one or two sports. Based on the answers, average time of sports participation per week was calculated. Finally, min/day of total physical activity (active transportation + sports participation) were included in the analysis.

**Sedentary behaviors **Screen time (i.e. TV and computer time) was assessed separately for weekdays and weekend days by two questions about time spent watching TV (including video and DVD) and computer activities. Mean TV, computer and total screen time per day were calculated. For the analyses, total min/day of screen time (TV watching plus computer use) were taken into account.

**Sleep duration **Child’s sleep habits reported by the parents included the number of hours the child sleeps per night on average, reported separately for weekdays and weekend days. For the purpose of this paper, only weekdays (hours/day of sleep duration) were taken into account as sleep during weekdays is likely to be more representative of usual sleep duration, due to the more regular bed- and get-up routine [13].

#### Parental education

Parents were asked to report their own level of education, as well as the level of education of the other parent/caregiver. The possible answers were; a) less than 7 years b) 7–9 years c) 10–11 years d) 12–13 years e) 14 years or more. These years take into account the different educational levels since preschool. Thus, the category “c” in this international dataset approximately distinguishes families with a caregiver who has completed medium or higher vocational, college or university training from other families. After preliminary analyses of the distribution of the variable, it was concluded that to recategorize into low, medium and high parental education level was not possible due to the small sample size included in the low category. Both scores (maternal and paternal education levels) were combined, and dichotomized into low (0, both parent/caregivers with fewer than 14 years of education) and high (at least one parent/caregiver with 14 or more years of education).

#### Anthropometric measurements

Body height and weight were measured by trained research assistants. The children were measured in light clothing without shoes. Body height was measured with Seca Leicester Portable stadiometer (to the nearest 0.1 cm). Weight was measured with a calibrated electronic scale SECA 861 (to the nearest 0.1 kg). Two readings of each measurement were performed. When readings differed more than 1%, a third reading was taken. Body Mass Index (BMI) and overweight status (overweight, obesity based on the International Obesity Task Force criteria (IOTF) [34] were calculated.

### Data analysis

To identify clusters with similar dietary, physical activity and sedentary habits, a combination of hierarchical and non-hierarchical clustering analysis was used [35]. Gender-specific cluster analyses were performed, due to the significant influence of gender in the EBRBs means (Table 1). Z-scores of all variables were calculated to standardize the data set before clustering. This prevents variables measured in larger ranges from contributing to the distance largely than variables with smaller ranges. Univariate and multivariate outliers (more than 3 standard deviations) were removed. In a first step, hierarchical cluster analysis was applied using Ward’s method, based on squared Euclidian distances [36]. At this stage, comparison of several possible cluster solutions was performed. Using the resulting centroids, a non-hierarchical k-means cluster analysis was performed, in order to further fine-tune the preliminary hierarchical cluster solution. ANOVA tests and post hoc Bonferroni tests were used to investigate the differences between each cluster on all indices.

**Table 1:** Gender-specific means (SD) of energy balance related behaviors in seven European countries participating in the ENERGY study

**Country**	**N**	**Sugared drinks (ml/day)**		**Physical activity (min/day)**		**Screentime (min/day)**		**Sleep duration (hours/day)**	
		**boys**	**girls**	**boys**	**girls**	**boys**	**girls**	**boys**	**girls**
Belgium	654	639(32.1)	597(28.1)	37(1.3)	37(1.2)	196(5.5)†	173(4.7)	9.6(0.04)	9.7(0.04)
Greece	958	409(17.9)†	329(12.7)	33(1.1)†	26(0.9)	214(5.0)†	177(3.6)	8.5(0.04)	8.5(0.04)
Hungary	811	843(35.9)†	735(29.0)	46(1.5)†	39(1.1)	231(5.7)†	194(4.7)	8.8(0.04)	8.9(0.04)
the Netherlands	362	844(47.7)	799(38.1)	41(1.6)†	35(1.5)	210(9.0)†	172(7.4)	9.6(0.06)	9.7(0.06)
Norway	750	482(23.6)†	323(16.1)	57(1.4)†	50(1.2)	197(5.1)†	162(4.2)	9.1(0.04)	9.1(0.04)
Slovenia	856	740(32.3)†	531(23.6)	48(1.3)†	42(1.2)	210(5.4)†	167(4.6)	9.0(0.04)	9.0(0.04)
Spain	893	465(21.5)†	331(16.1)	44(1.2)†	30(1.0)	193(4.9)†	158(4.0)	9.2(0.04)	9.2(0.04)
Total	5284	606(11.4)†	490(9.1)	44(0.5)†	36(0.5)	207(2.1)†	172(1.7)	9.1(0.01)	9.1(0.01)

† Significant differences between gender p < 0.05.

Chi-square tests were performed to investigate the differences on cluster distribution by country, BMI category, and parental education level. Odds ratios for being allocated in one specific cluster by parental education level were also calculated (adjusting for age, country and BMI z-scores).

All statistical analyses were performed using the Predictive Analytic Software (PASW) version 18.0 (SPSS inc., Chicago, IL, USA).

## Results

Table 2 describes the characteristics of the participating children (n = 5284). The mean age of children was 11.6 (0.73) years, 54.3% girls. As defined by at least one parent/caregiver with at least 14 years of education, 67.5% of the participants were included in the high parental education group. Across the countries, 20.4% and 4.8% of children were overweight (including obese) and obese respectively. The sample size varied between countries, mostly because of differences in parental response rates.

**Table 2:** Characteristics of the total sample

		**Mean (SD)**
Age		11,6 (0.73)
		
Gender		N (%)
	Boys	2413 (45.7)
	Girls	2871 (54.3)
BMI status		
	Normal weight	3930 (74.4)
	Overweight	1030 (20.4)
	Obese	248 (4.8)
Parental education level		
	Low	1686 (32.5)
	High	3497 (67.5)
Country		
	Belgium	654 (12.4)
	Greece	958 (18.1)
	Hungary	811 (15.3)
	the Netherlands	362 (6.9)
	Norway	750 (14.2)
	Slovenia	856 (16.2)
	Spain	893 (16.9)

Based on the four EBRBs indices, the five-cluster solutions were found to be adequate and meaningful regarding the different patterns for both genders, but the clusters differed somewhat between boys and girls. The reliability and stability of the created five-cluster solutions were examined by randomly dividing the samples into two subsamples, in which the same clustering procedure was repeated. Kappa degrees of concordance in cluster membership were calculated by comparing membership of both subsamples separately with these of the total gender-specific sample, showing excellent agreement for both girls (k = 0.97 and 0.98 for the first and second subsample respectively), and boys (k = 0.98 and 0.97 for the two subsamples).

### Description of the clusters

**Figure 1:**
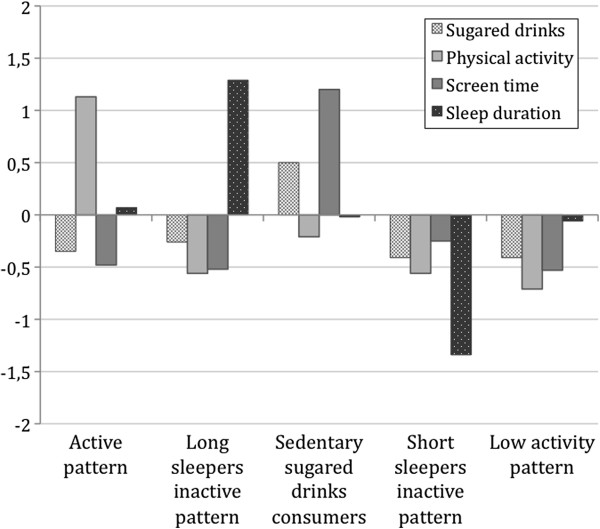
Standard deviation scores of clusters on energy balance-related behaviors for girls

**Figure 2:**
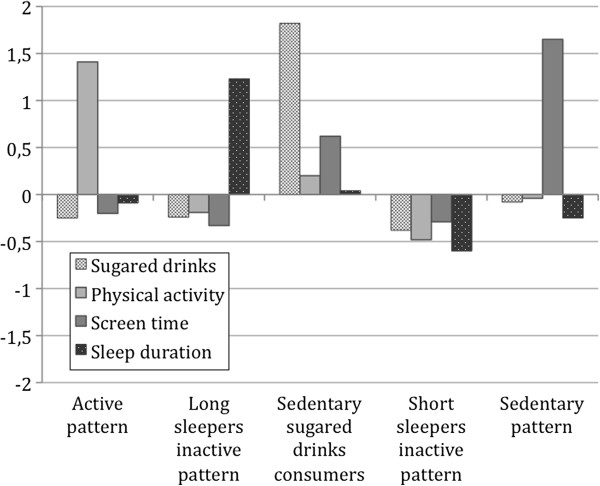
Standard deviation scores of clusters on energy balance-related behaviors for boys

Four of the five clusters received the same labels for boys and girls due to the similar characteristics for both sexes, although cluster mean values differed. These four clusters were labeled as active pattern; long sleepers inactive pattern; sedentary sugared drinks consumers; short sleepers inactive pattern. The fifth cluster differed between girls and boys and was labeled as “low activity pattern” for girls and as “sedentary pattern” for boys. Distinguishing characteristics of each cluster are indicated by high or low z-scores. Final cluster centers and labels are presented in Figures 1 and 2. Row mean values (mean ± standard deviation) are presented in Table 3.

**Table 3:** EBRBs in the final clusters (absolute values), ANOVA and results of Bonferroni test

	**Active pattern**	**Long sleepers inactive pattern**	**Sedentary sugared drinks consumers**	**Short sleepers inactive pattern**	**Low activity/ Sedentary pattern**	***F***
**GIRLS**	n = 641	n = 615	n = 436	n = 529	n = 650	
**Sugared drinks**	408 ± 15.6 ^a †^	467 ± 18.0 ^a^	975 ± 30.9 ^b ††^	367 ± 15.6 ^a^	367 ± 13.9 ^a ††^	161.416*
X ± SE (ml/day)						
**Physical activity**	69 ± 0.6 ^a ††^	26 ± 0.6 ^c ††^	34 ± 1.0 ^d ††^	26 ± 0.7 ^d †^	22 ± 0.5 ^b ††^	892.090*
X ± SE (min/day)						
**Screen time**						578.650*
X ± SE (min/day)	143 ± 2.6 ^a ††^	140 ± 2.7 ^a ††^	316 ± 3.6 ^b ††^	168 ± 3.2 ^c^	138 ± 2.3 ^a ††^	
**Sleep duration**	9.1 ± 0.02^a ††^	10.2 ± 0.02^c †^	9.0 ± 0.03	7.9 ± 0.01 ^e ††^	9.0 ± 0.00 ^b ††^	1841.197*
X ± SE (hours/day)						
**BOYS**	n = 540	n = 479	n = 240	n = 753	n = 401	
**Sugared drinks**	476 ± 16.0 ^b^	482 ± 17.9 ^d^	1858 ± 27.0 ^e^	390 ± 11.3 ^c^	586 ± 19.8 ^a^	791.487*
X ± SE (ml/day)						
**Physical activity**	76 ± 0.7 ^b^	35 ± 0.8 ^d^	45 ± 1.6 ^e^	28 ± 0.6 ^c^	39 ± 1.0 ^a^	596.419*
X ± SE (min/day)						
**Screen time**	173 ± 3.2 ^b^	160 ± 3.3 ^d^	256 ± 6.4 ^e^	163 ± 2.3 ^c^	362 ± 3.2 ^a^	646.535*
X ± SE (min/day)						
**Sleep duration**	9.0 ± 0.03 ^b^	10.1 ± 0.02 ^d^	9.1 ± 0.05 ^e^	8.5 ± 0.02 ^c^	8.8 ± 0.04 ^a^	492.828*
X ± SE (hours/day)						

^a,b,c,d,e ^Significant differences. A cluster mean is significantly different from another mean if they have different capitals. * *P* < 0.001.

^† ^Significant differences between gender p < 0.05.

^†† ^Significant differences between gender p < 0.001.

The active pattern was characterized by z-scores above 0 for physical activity and z-scores below 0 for sugared drinks consumption and screen time. The physical activity score was significantly higher in the boys’ cluster (z-score = 1.41) compared to the girls’ cluster (z-score = 1.13). The long sleepers inactive pattern cluster was characterized by high values on sleep duration (z-scores >1.23) and low scores on the rest of behaviors. The sedentary sugared drinks consumers group presented high values for sugared drinks consumption and for screen time. However, the sugared drinks score was much higher in the boys’ cluster (z-score = 1.82) compared to the girls’ cluster (z-score = 0.50), while the screen time score was higher in the girls’ cluster (z-score = 1.20) compared to the boys’ cluster (z-score = 0.62). The short sleepers inactive pattern cluster showed very low values for sleep duration and low values on the rest of behaviors. Girls included in this cluster presented lower sleep duration values (z-score = -1.34) compared to boys (z-score = -0.60). Low activity pattern cluster in girls was characterized by low values on all the behaviors, while the sedentary pattern cluster in boys presented a very high score on screen time (z-score = 1.65) and almost average values for the rest of the behaviors.

### Cluster group characteristics

The associations between the five clusters and the socio-demographic variables (country, BMI and parental education level) are presented in Table 4. Significant chi-squares were found for country, BMI and parental education level.

**Table 4:** Percentage of girls and boys in each cluster, according to socio-demographic factors

**GIRLS**								**BOYS**						
	**N**	**Active pattern**	**Long sleepers inactive pattern**	**Sedentary sugared drinks consumers**	**Short sleepers inactive pattern**	**Low activity**	**Chi-square**	**N**	**Active pattern**	**Long sleepers inactive pattern**	**Sedentary sugared drinks consumers**	**Short sleepers inactive pattern**	**Sedentary pattern**	**Chi-square**
**Country**		(%)	(%)	(%)	(%)	(%)			(%)	(%)	(%)	(%)	(%)	
Belgium	356	8.6	27.5	15.1	3.0	7.7	923.297*	298	5.9	30.5	13.8	7.2	8.2	606.361*
Greece	532	7.8	8.3	11.0	46.5	21.1		426	7.6	7.1	5.0	31.9	24.7	
Hungary	458	16.1	7.2	28.7	17.0	14.8		353	16.5	6.5	28.8	12.9	16.7	
the Netherlands	190	3.3	15.6	8.5	1.5	4.3		172	3.7	12.9	13.3	4.1	6.7	
Norway	404	28.9	9.3	7.6	8.3	13.1		346	28.9	10.2	7.1	9.8	12.5	
Slovenia	463	24.2	10.4	18.6	13.0	14.5		393	19.6	11.7	24.6	15.4	14.0	
Spain	468	11.2	21.8	10.6	10.6	24.6		425	17.8	21.1	7.5	18.7	17.2	
Total	2871							2413						
**BMI**														
Normal weight	2198	82.4	85.7	74.9	68.8	74.6	78.338*	1732	80.9	78.7	74.8	64.9	68.6	55.800*
Overweight	509	15.5	12.0	19.1	22.4	21.7		521	16.4	17.3	20.1	27.7	24.8	
Obese	122	2.1	2.3	5.9	8.7	3.7		126	2.6	4.0	5.1	7.4	6.6	
**Parental education**														
Low	820	24.4	28.2	42.4	42.4	30.0	64.283*	665	25.3	26.0	39.2	32.7	38.8	29.133*
High	1713	75.6	71.8	57.6	57.6	70.0		1461	74.7	74.0	60.8	67.3	61.2	

* *P* < 0.001.

Country specific representation varied in the different clusters. The active pattern cluster comprised significantly more Norwegian and Slovenian boys and girls; the long sleepers inactive cluster included the highest proportion of Belgian participants, while the short sleepers inactive pattern included the highest proportion of Greek boys and girls; the sedentary sugared drinks consumers were more prevalent in the Hungarian sample and, finally, the fifth cluster was mainly represented by Spanish and Greek girls (low activity pattern), and by Greek and Spanish boys (sedentary pattern).

The long sleepers inactive pattern had the lowest proportions of overweight and obese girls (12%; 2%), while the active pattern had the lowest proportions of overweight and obese boys (16%; 3%). The short sleepers inactive pattern comprised the highest proportion of overweight and obese girls (22%; 9%) as well as boys (28%; 7%).

The active pattern and long sleepers inactive pattern clusters showed the highest proportion of participants with parental (76% and 72% for girls; 75% and 74% for boys) education level. The sedentary sugared drinks consumers cluster and the short sleepers inactive pattern cluster comprised the highest proportions of girls with lower parental (42% and 42%) educational level, while the sedentary pattern and the sedentary sugared drinks consumers clusters presented the highest proportion of boys with lower parental (39% and 39%) educational level.

After exploring the associations of gender, country and BMI with the cluster distribution, odds ratios were calculated for being allocated in a specific cluster by parental education level, adjusted for the other socio-demographic characteristics (Table 5). The results show that girls (OR: 0.58; 95% CI: 0.46-0.74) and boys (OR: 0.69; 95% CI: 0.54-0.87) with lower educated parents were significantly less likely to be allocated in the active pattern. Girls (OR: 0.78; 95% CI: 0.65-0.94) and boys (OR: 0.69; 95% CI: 0.55-0.87) with lower educated parents were also less likely to be allocated in the short sleepers inactive pattern. On the contrary, girls (OR: 1.93; 95% CI: 1.40-2.66) and boys (OR: 1.45; 95% CI: 1.09-1.92) with lower educated parents were more likely to be allocated in the low activity/sedentary pattern. Finally, girls with lower educated parents were more likely to be allocated in the long sleepers inactive pattern (OR: 1.37; CI: 1.07-1.76) and in the sedentary sugared drinks consumers pattern (OR: 1.35; 95% CI: 1.08-1.70).

**Table 5:** Odds ratios (OR) and 95% confidence intervals (CI) for being allocated in a specific cluster by parental education levels (Ref: High; adjusted by country, BMI z-scores and age) for girls and boys

	**Low parental education**	
**Girls (n = 3025)**	**OR**	**CI**
Active pattern	0.58†	0.46-0.74
Long sleepers inactive pattern	1.37*	1.07-1.76
Sedentary sugared drinks consumers	1.35*	1.08-1.70
Short sleepers inactive pattern	0.78*	0.65-0.94
Low activity pattern	1.93†	1.40-2.66
	
**Boys (n = 2604)**		
Active pattern	0.69†	0.54-0.87
Long sleepers inactive pattern	1.24	0.99-1.56
Sedentary sugared drinks consumers	0.96	0.74-1.27
Short sleepers inactive pattern	0.69*	0.55-0.87
Sedentary pattern	1.45*	1.09-1.92

*p < 0.05;†*p* < 0.01.

## Discussion

The first goal of this paper was to explore the existence of clusters of EBRBs in a large sample of school-age children across Europe. Five reliable EBRBs clusters showing good stability were identified for both boys and girls. Meaningful clusters were found while correlations between the EBRBs were low, showing that low correlations do not exclude co-occurrence of health-related behavioral indicators within certain groups. One of the major findings is that none of the clusters showed marked healthy or unhealthy trends for all the included EBRBs. This fact implies that health-related behaviors do not always discriminate in the same direction. Children with specific healthy habits are not necessarily predisposed to be involved in other specific healthy behaviors. Similar results were found in previously published studies [37,38]. Cluster prevalence was not equal for each subgroup; the most prevalent were patterns characterized by low physical activity.

The results pertaining to the second goal – the characterization of the cluster solutions by parental education level and correlates – revealed that the cluster distribution was significantly different according to parental education level. It was clear that parental education was relevant for children’s EBRBs patterns. Children from higher educated parents were more likely to be allocated in the active pattern cluster, while low activity/sedentary pattern and sedentary sugared drink consumers were more prevalent among children from lower educated parents. It is noteworthy that low activity/sedentary pattern and sedentary sugared drink consumers pattern combined unhealthy levels in more than one of the assessed EBRB. These results suggest that children from lower educated parents are not only more likely to engage in less healthy behaviors [39,40], but also more prevalent in clusters combining more unhealthy lifestyles. These results suggest the need to specifically address the relevance of physical activity and sedentary behaviors in obesity prevention strategies focusing on lower educated parents and their children.

The results show that children (both boys and girls) in the active cluster had below average screen time levels, and those children in the sedentary clusters (sedentary sugared drinks consumers for girls, sedentary pattern for boys) had also below average physical activity levels. This may suggest that there is some displacement between sedentary behavior and physical activity, and vice versa, although earlier studies suggest being careful with this displacement theory, as this displacement mechanism seems not to be universal across countries [41,42].

The cluster solutions included two groups mainly characterized by their sleep duration scores (Long sleepers inactive pattern and short sleepers inactive pattern). Even if these groups showed an association with parental education, sleep did appear to differ in a stronger way according to country with more long sleepers in northern countries and more short sleepers in southern-east countries, in line with previous studies [13]. This distribution seems to reflect an important country-specific cultural influence on sleep duration in children.

The cluster solutions showed large gender differences. These differences were reflected not only in the clustering itself (with a gender specific cluster, namely the low activity/sedentary pattern cluster) but also in the behavioral levels in the rest of the clusters. One of the main differences was found for sugared drinks intake, much higher for boys compared to girls, in agreement with previous literature [43,44].

Additionally, cluster solutions were also characterized by BMI, being the short sleepers inactive pattern cluster the one with the highest proportion of overweight and obese boys and girls. In general, previous research showed that short sleep duration is associated with higher risk of childhood obesity [26,45]. However, our findings should be interpreted with caution, taking into account the disproportionate country-specific representation in the short sleepers inactive cluster (i.e. Greek sample).

Finally, it has to be kept in mind that this study is subject to some limitations. First, this concerns a cross-sectional study providing evidence for associations but not causation. Further, data on dietary, physical activity and sedentary behaviors were based on self-reports, and thus possibly biased. However, the measures showed good test-retest reliability and construct validity [33]. Additionally, for some behaviors both 24-h recall and frequency questions were included, showing similar results. When considering sedentary behaviors it is also important to note that some sedentary activities, like reading or studying were not included in the present study. Is therefore possible that questionnaires did not reflect the real, total time spent in sedentary behaviors.

Strengths of the present study include the large multinational sample from different regions across Europe and the standardized data collection protocol across the different countries. The use of cluster analysis for the assessment of EBRBs in relation with socio-demographic variables allowed us to reflect a more ecological view of the actual children’s behaviors and their socio demographic correlates. The described clusters in the present analysis showed a good stability and could therefore be seen as representative clusters for European children. It could be interesting for future research to examine the effectiveness of tailored obesity prevention strategies focusing on the most prevalent combinations of unhealthy EBRBs, with special attention to subgroups at higher risk, like children of lower educated parents.

## Conclusions

The obtained stable cluster solutions allowed us to classify children according to several health behaviors that are associated with risk for overweight and obesity. None of the clusters showed marked healthy or unhealthy trends for all the included EBRBs. However, parental education was associated with the odds for being allocated in specific clusters, mainly characterized by their physical activity and screen time scores that combined more than one unhealthy EBRB. Children with lower educated parents seemed to be more likely to present unhealthier EBRBs clustering compared to those with highly educated parents. Therefore, special attention should be given to lower educated parents and their children when developing childhood obesity prevention strategies focusing on clusters of unhealthy lifestyles.

## Competing interests

The authors declare that they have no competing interests.

## Authors’ contribution

JB and ASS developed the measurement instruments. ASS developed the study protocol, coordinated and supervised the international data collection. ASS, IDB, FNV, YM, EK, NJ and JMFA contributed to or supervised the national data collection procedures. JMFA conducted the data analyses under supervision of IDB and LAM. JMFA drafted the manuscript. All authors read and approved the final manuscript.
